# A patient-centric distribution architecture for medical image sharing

**DOI:** 10.1186/2047-2501-1-3

**Published:** 2013-01-10

**Authors:** Liviu Constantinescu, Jinman Kim, Ashnil Kumar, Daiki Haraguchi, Lingfeng Wen, Dagan Feng

**Affiliations:** 1 School of Information Technologies, University of Sydney, Building J12, Sydney, Australia; 2 Department of PET and Nuclear Medicine, Royal Prince Alfred Hospital, Sydney, Australia

**Keywords:** Telemedicine, Medical image viewer, patient-centric healthcare, Clinical workflow support system, Positron emission tomography - computed tomography

## Abstract

Over the past decade, rapid development of imaging technologies has resulted in the introduction of improved imaging devices, such as multi-modality scanners that produce combined positron emission tomography-computed tomography (PET-CT) images. The adoption of picture archiving and communication systems (PACS) in hospitals have dramatically improved the ability to digitally share medical image studies via portable storage, mobile devices and the Internet. This has in turn led to increased productivity, greater flexibility, and improved communication between hospital staff, referring physicians, and outpatients. However, many of these sharing and viewing capabilities are limited to proprietary vendor-specific applications. Furthermore, there are still interoperability and deployment issues which reduce the rate of adoption of such technologies, thus leaving many stakeholders, particularly outpatients and referring physicians, with access to only traditional still images with no ability to view or interpret the data in full. In this paper, we present a distribution architecture for medical image display across numerous devices and media, which uses a preprocessor and an in-built networking framework to improve compatibility and promote greater accessibility of medical data. Our INVOLVE2 system consists of three main software modules: 1) a preprocessor, which collates and converts imaging studies into a compressed and distributable format; 2) a PACS-compatible workflow for self-managing distribution of medical data, e.g. via CD USB, network etc; 3) support for potential mobile and web-based data access. The focus of this study was on cultivating patient-centric care, by allowing outpatient users to comfortably access and interpret their own data. As such, the image viewing software included on our cross-platform CDs was designed with a simple and intuitive user-interface (UI) for use by outpatients and referring physicians. Furthermore, digital image access via mobile devices or web-based access enables users to engage with their data in a convenient and user-friendly way. We evaluated the INVOLVE2 system using a pilot deployment in a hospital environment.

## Background

Medical imaging has become an important component in modern medicine by providing non-invasive anatomical or functional information. It has been widely used in the clinical management of oncology such as initial diagnosis, staging and re-staging, treatment planning, and assessment of treatment response. Hybrid multi-modality imaging devices, combining positron emission tomography with computed tomography (PET-CT) or magnetic resonance imaging (PET-MR), are capable of acquiring two complementary images in a single session, and have delivered improved imaging outcomes for patients. For example, PET-CT has been shown to improve cancer diagnosis, localization, and staging compared to single modality PET or CT alone [1–3]. Such medical images are stored and transmitted, alongside electronic medical records and reporting information, by Picture Archiving and Communication Systems (PACS) [4]. These systems collectively form a transmission network consisting of imaging devices, computer workstations for interpreting images, and archival systems for images and reports. These storage and transmission systems make use of a format called Digital Imaging and Communications in Medicine (DICOM) [5], the dominant standard for image storage, image query and data transfer to and from PACS [6].

However, file size and compatibility issues still limit the distribution of medical imaging: for instance, the size of a typical whole-body PET-CT study will vary between 160 Mb and 240 Mb in size, with some studies markedly larger depending on the type of study and the resolution of the scanning hardware. To solve this, it has become increasingly popular to have patients carry a CD/DVD disc of imaging data back to their referring physicians. However, as discussed below, such systems remain limited in their functionality and capabilities, and do not fully address the needs of the patient and referring physician populations.

The push for patient-centric and participatory healthcare [7] requires that patients should be active participants in their ongoing care; and this necessitates that they be given direct access to, and an understanding of, the imaging data that underlies their physician’s decision-making process [8]. The popularity of online Personal Health Record management systems such as Microsoft HealthVault [9] is direct evidence that patients value the opportunity to personally view their medical records, and share information with family, other practitioners, and their peers [10]. Further, the literature also shows that the current delays between image acquisition and the communication of results is considered unsatisfactory by most patients [11], indicating that any improvements to this workflow would likely result in a rise in patient satisfaction. Currently, patients undergo a lengthy and emotionally difficult wait, even in the case where they literally have the information in-hand in the form of a CD/DVD. This wait can be reduced via automation or elimination of manual processes, to reduce delays, or via networking and data portability features that connect patients to caregivers and information, to speed communication of results. Further, the above study noted that patients have no strong preference as to which physician interprets this information and how. This presents an opportunity to reduce radiologists’ isolation from patients by having them fulfil their reporting role directly, using the software as an intermediary. This could have significant benefits for patient understanding and the radiology specialty as a whole [12]. However, this also requires that the intermediary software be incorporated into the hospital’s clinical workflow, allowing it to be accessible (and useful) to radiology specialists and outpatients alike.

Many referring physicians (young doctors especially) also show a strong preference for direct access to PACS data and to hospital colleagues: when ordering a radiological study, they would prefer to be able to “call u” the images immediately and have the chance to discuss the case collaboratively with the radiologist, rather than receiving a simple textual report and a standalone DICOM viewer on a disc [13]. Similarly, in intra-hospital or emergency cases, delivering radiology studies to the right person on time can be critical, necessitating an informatics-based distribution approach [14].

Though the noted issues of file size and compatibility (alongside other factors such as security and privacy) make physical distribution an attractive option, the above circumstances posit the need for flexibility in this scenario. We propose that the necessary solution is a convenient, full-featured medical image deployment platform that supports physical sharing, but is optimised to also operate effectively across the Internet or in a browser, usable on the widest possible range of consumer hardware, designed for operation by untrained users, and capable of networked distribution of medical imagery when necessary.

### Related work

Presently available systems do not meet all of these criteria, and can be generally divided into two categories:

Proprietary systems such as Codonics Virtua^*®*^[15] or Medigration’s MediImage [16] are a good baseline, producing (often platform-specific) standalone CDs/DVDs. Clinicians need only transfer a study via DICOM, and then physically hand the resulting disc to the patient prior to the end of their visit. Relying entirely on physical media, however, this approach sacrifices many of the benefits of digital imaging, offering few advantages over paper/film records when it comes to distribution. More full-featured proprietary systems such as MIM / Mobile MIM / MIM Cloud [17], PeerVue’s QICS [18] and Siemens’ syngo^*®*^ Webspace [19] offer a much wider range of distribution and sharing features but centralise their offering around that vendor’s own system. This is easier for vendors to implement, as there is no need for compatibility with or integration into any workflow but the company’s own, but the transition cost for hospitals is high. Further, outpatients and referrers usually cannot access the full benefits of the system, which is located within the hospital.

The second category involves open solutions, often outpatient-focused, such as DICOM Works [20], Sante viewer [21], AMIDE [22], ezDICOM [23], etc. These systems, viewers and tools help to promote innovation and the adoption of standards in the field, but are typically very limited in their functionality and no true substitute for vendor systems [24]. Even relatively full-featured systems such as OsiriX [25] have interfaces that are complex and physician-focused, making them unsuitable for outpatient use. Likewise, they often provide limited support for networked use or the communication of results, because this is not traditionally a function of the radiological viewer. Finally, due to the limitations of consumer devices, access speeds can sometimes be a significant factor limiting the outpatient-usability of software in either category.

### Contributions

In this paper, we present INVOLVE2: a patient-centric distribution system that was designed from the ground up to address these issues. INVOLVE, which stands for **I**nteractive **N**etworked **VOL**ume **V**isualization **E**nvironment, makes use of a powerful preprocessor and advanced automated networking components developed in-house to rapidly deploy PET-CT studies across a variety of digital channels and to mobile devices.

INVOLVE2 is the culmination of our ongoing research into patient-centric and participatory healthcare. The INVOLVE2 architecture combines a number of novel medical imaging technologies developed by our research group into a distribution system optimised for convenient use by outpatients, referrers and hospitals alike. Major components of INVOLVE2 include parts of the multi-modality INVOLVE (v1) viewer [26], the TAGIGEN [27] online medical image comparison system, and an implementation of the SparkMed shared data model for medical data integration and mobile healthcare data delivery [28]. Mobile distribution in INVOLVE2 is based on prototypes developed for SparkMed [29, 30] and the VacTube [31] browser widget.

## Methods

**Figure 1:**
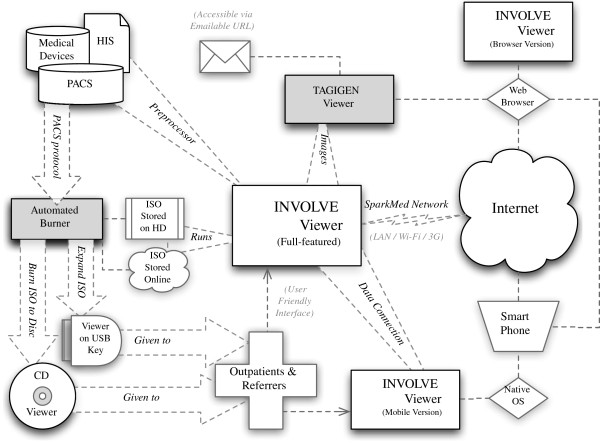
**Overall architecture for the Interactive Networked VOLume Visualization Environment (INVOLVE2), showing (from left to right) the main modules, communication layers and submodules of the system.**

The architecture of INVOLVE2 is shown in Figure 1. The system has three modular components, which combine to form a unique workflow. The first of these is the INVOLVE2 viewer application, an intuitive, human-readable image viewer designed with outpatients and referrers in mind, which supports live collaboration and radiologist-reporting directly within the app. The second component is a preprocessor which automates the process of creating patient-study CDs, and performs numerous rendering and preparatory operations to allow all of our other components to load faster and stream data more effectively. Finally, our third component is a suite of mobile and web applications which automatically connect with one another and support browser-based or mobile distribution of medical imagery and radiologist’s reports. These are networked solutions, and connect to the central software over the network.

The diagram further shows PACS, RIS/HIS (Healthcare Information Systems) and related DICOM-compatible systems as data sources for the viewer. Due to its powerful preprocessor, INVOLVE2 can accept data directly from these sources for use with the INVOLVE2 Viewer, or the PACS’ own protocols can be used to transmit patient studies to the INVOLVE2 Burner, which will likewise use the preprocessor: this time to generate an INVOLVE2 CD with all of these capabilities, for passing on to patients (via CD or USB key) or for direct use either saved to disk or online.

### Preprocessor

The INVOLVE2 Preprocessor performs a sequence of compression, transcoding and rendering operations to make standard DICOM-based medical datasets compatible with the variety of fast-loading, networking and browser-based techniques used by the INVOLVE2 components.

**Figure 2:**
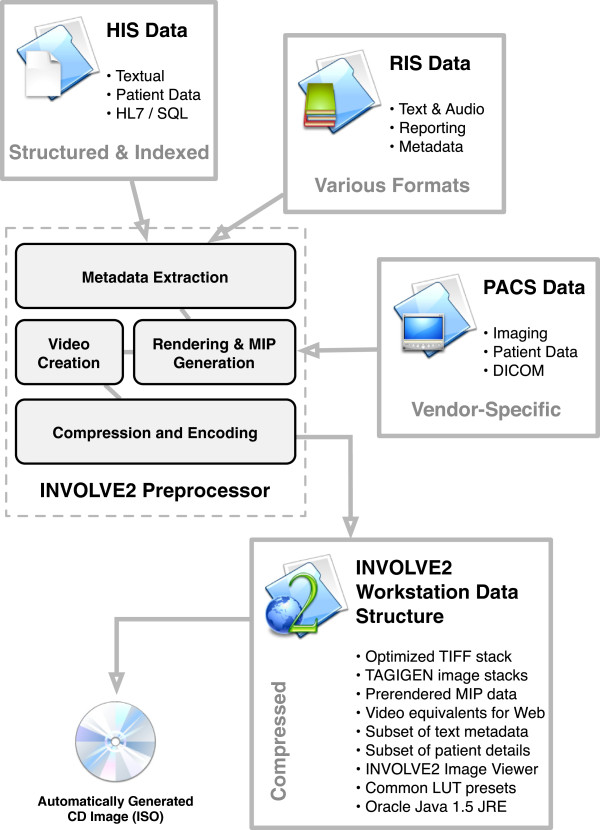
**Functionality of the INVOLVE2 Preprocessor module.**

A flow chart showing the design, input and output of our back-end preprocessor system is depicted in Figure 2. To reduce data size but facilitate rapid loading, the DICOM source files are converted into a TIFF stack, with the necessary textual and meta-data stored alongside the stack separately. We also reslice each modality into two pre-rendered views (coronal and sagittal), and also render a colorized fusion view, to create a set of JPEG stacks for use by TAGIGEN. These secondary stacks are not required by the INVOLVE2 Viewer, which generates the other views on-the-fly by pixel processing. We also generate a coronal Maximum Intensity Projection (MIP) of the PET image, stored in TIFF format. Finally, numerous compressed thumbnails are generated for use by TAGIGEN. The file-size ratio of a fully-preprocessed set of INVOLVE2 stacks (including rotational MIP and TAGIGEN data) to the source DICOM is roughly 1:1.86 (a file-size decrease of just under 50%). All of our data are stored primarily in lossless TIFF format, to ensure diagnostic usability, with secondary datasets such as TAGIGEN’s only used to optimise render speeds on certain platforms and mobile devices.

Some applications of INVOLVE2 data, in particular pre-rendering of the 3D MIP data and streaming stacks to mobile hosts, have greater compression requirements, and live transcoding needs to be done by our network distribution architecture in order to adapt to the device’s processing capability and available bandwidth. When transmitting to web-only devices, our preprocessor uses FLV encoding (On2 VP6) for its superior compression capability. The compression ratio of the original INVOLVE2 image stack to a compressed FLV stack varies based on the encoder settings used, but averages at approximately 1:3.85 (a decrease of 74%).

#### Automated burner

While the preprocessor can be used to process raw data for use with the INVOLVE2 system, a subsystem thereof is designed to bundle the preprocessed data with a copy of the INVOLVE2 software for distribution. This Automated Burner component generates a standalone CD Image (ISO), which can be burned to disc, saved to one’s hard-drive or distributed via USB key. The software contained on this CD image has no installation requirements and runs equally well on Windows, Unix and Mac OS X.

In order to create a self-contained INVOLVE2 viewer for use by outpatients, referrers and other stakeholders, all that is necessary is for the INVOLVE2 CD image file to be burned to CD-ROM or expanded into the root directory of a USB key. The Automated Burner is capable of accepting PET-CT studies (in DICOM format) from any standard hospital PACS server. These studies are queued up in an automatic list, and sequentially processed - one patient to each CD image - alongside all of the relevant reporting and meta-data. A full specification of what is included on the CD image INVOLVE2 generates is given in Figure 2.

There is minimal need for human interaction in the burning process, requiring only that blank CDs be provided upon request. Whereas we have implemented it as a dedicated machine, the Automated Burner is cross-platform^a^ and low in system requirements, requiring only a CD burner to run.

### Viewer

The INVOLVE2 viewer is able to run on every major desktop operating system without installation and hence is fully compatible with a wide range of modern personal computers. Further, the application has a number of built-in networking technologies which allow it to transmit its views and metadata over TCP/IP or UDP in order to communicate with mobile hosts.

Multi-modality medical images such as PET-CT require specialized viewing software with a specific range of imaging features to effectively assimilate: interpretation of any given scan relies on image processing adjustments to the orthogonal views such as window/level transforms, changes to the images’ dynamic range, the application of colour lookup-tables, and adjustment of the fusion ratio [32]. We chose to implement our cross-platform viewer software using Java 1.5. Due to this choice, we based the majority of our image processing tasks on the ImageJ package [33]. This library was used for all of the viewer’s image processing needs, including preprocessing.

In order to design a patient-centric viewer, we elicited the requirements for our image viewing software using an iterative design process in collaboration with physicians, so as to design information displays that would be highly informative and useful for both patients and radiologists alike. This was similar to the approach followed in [34]. We elicited the following requirements:

1. When the viewer is started, it should load and render the images in less than a minute.
2. The viewer should switch between views (projections along a different axis) in less than a minute.
3. The viewer should display two aligned slices (one from PET and one from CT).
4. The viewer should allow the PET slice to be replaced with a fused slice.
5. The viewer should display a stoppable, auto-rotating MIP of the PET data.
6. The viewer should provide the following controls:
  - Navigation for the aligned slices and MIP.
  - Toggles for switching between coronal and sagittal views.
  - Toggles for switching between normal and fused images.
  - Fusion ratio adjustment.
  - Colour table switching for fused views.
  - Patient information display.
7. All controls should be labelled using simple English (not medical jargon) that is understandable by a layperson.

#### User interface

**Figure 3:**
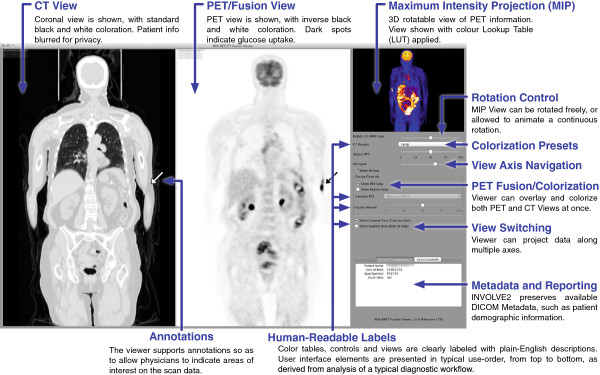
**The user interface of our INVOLVE viewer, shown running under Mac OS X Lion, annotated with image functionality.**

Our viewer application is built on a modular system, allowing it to be equipped with a series of generic views and controls in a customisable configuration. Our medical image viewer’s standard interface is optimised for PET-CT data display and supports full navigation, colour look-up tables (LUT) and window/level transforms, as well as multi-modality fusion and simple annotation (such as using small arrows to draw attention to particular parts). The interface shown in Figure 3 is this PET-CT interface, and other setups (for brain imaging, advanced users, etc.) are possible using different configurations of the same series of INVOLVE2 views. The two side-by-side image panels display the CT and PET images; controls can be used to swap the PET image with a fused PET-CT image. The image panel in the top right corner displays the rotating MIP. These image displays and controls satisfy Requirements 3, 4, 5 and 6.

We developed a patient-centric UI, per Requirement 7, where all components were ordered according to the sequence of expected usage (top-to-bottom) and labelled with terms a layperson would understand. Four predefined window/level presets (lung, abdomen, brain and bone) were provided to allow simple emphasis of anatomical structures without the user needing to specify numerical window width and window level values. Likewise, colour lookup-tables (LUTs) are presented such that hospital-specific terms are substituted with simple textual descriptions. The coronal and sagittal view toggles were renamed to describe the cross-section angle from the user’s point of view (front-to-back and side-to-side). All of this component labelling is visible in Figure 3.

### TAGIGEN viewer

**Figure 4:**
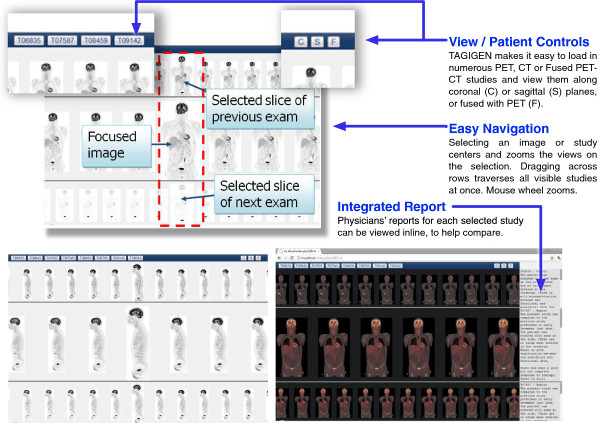
**The user interface of our TAGIGEN viewer, which allows multiple PET-CT studies to be combined, compared and correlated.**

TAGIGEN, shown in Figure 4, is a web-based image viewer with dynamic functionality that allows for the rapid display of massive data sets of multi-modal and temporal PET-CT images. Such browser-based, client-server solutions are an effective, low-overhead means of allowing a wide range of users to access all of the functionality and benefits of the INVOLVE2 system.

The TAGIGEN viewer is a fast, powerful and interactive image viewer with an array of specialized features designed to take advantage of the INVOLVE2 preprocessor by harnessing its capabilities in a different context: namely the temporal navigation of and comparison of multiple PET-CT datasets for the purpose of staging and computer aided diagnosis. Its capabilities include dynamic retrieval, multi-modal retrieval and sophisticated web-based medical image navigation. This viewer displays the complete image history of selected patients on a single web page, allowing for direct visual comparison and intuitive drill-down-style navigation so as to isolate, compare and stage key features.

This system is a useful and practical subcomponent of the INVOLVE2 architecture, and exemplary of the kind of specialised applications that can be created using the INVOLVE2 preprocessor, networking and browser-based components. TAGIGEN has undergone user trials to determine its practicality and fitness-for-purpose, and initial results have shown it to be effective at visualising patient data over time (even in the case of untrained users), and indicate that it is easy to use (respondents rated its usability at an average of 4 out of 5). Its tools and views represent a practical minimum for the purpose it serves, and it is capable of interfacing with the main INVOLVE viewer in order to provide more in-depth navigability where necessary. As such, it also serves as a powerful navigational component for the INVOLVE2 system itself.

### Mobile access

A subset of the INVOLVE2 Viewer’s functionality also runs on mobile devices - either as a web-based Rich Internet Application or natively on the Apple iOS. Due to its use of distributed user interface objects and automated networking, the INVOLVE2 Viewer is able to automatically synchronise with these and other desktop-based INVOLVE2 Viewers, sharing image data and application state. This enables collaboration and interactive remote display of the study data over great distances. Whereas a number of mobile medical image viewer applications already exist [35–38], the INVOLVE2 approach is unique in its flexibility, remote control capability and compatibility.

The networking subsystem of INVOLVE2 is generated automatically by SparkMed (for the details of which, please see [28]). This network layer operates independently and intelligently to deliver timely and error-free data to interfaces running on all compatible devices within its network, affording each respective INVOLVE subsystem running on that network a secure and trustworthy link back to its main INVOLVE2 viewer. As such, deploying the INVOLVE2 viewer within a home or referring physician’s network acts as a catalyst which allows mobile and browser-based INVOLVE systems to also run within that environment (though they can also be deployed over the Internet). We achieve this through the automatic generation and maintenance of a peer-to-peer overlay network that straddles the existing network topology desktop systems and handheld devices. In so doing, our system provides a model for how to meet the challenge of medical data integration for mobile medical image deployment without the need for migration to proprietary systems or extensive redevelopment. All that is needed to support mobile and web deployment is to run the INVOLVE2 viewer somewhere.

This approach also increases the functional expandability of the overall INVOLVE2 system by allowing for easy integration with other third-party applications which implement the same kind of simple interprocess communication techniques. Such browser-server type solutions are an effective, low-overhead means of allowing other developers to access all of the functionality and benefits of INVOLVE2 while focusing on different interests [39].

## Results

There were three main technical goals in designing and developing the INVOLVE2 Viewer. The first was performance optimisation, including reduced load times, speed-ups due to pre-loading of images and effective use and image navigation even on very limited consumer PCs or over the web. We also focused on designing remote capability, to allow for instant deployment and advanced uses such as Smartphone-based collaboration. Finally, we required that INVOLVE2 have the ability to generate fully functional INVOLVE2 systems for distribution via CDs or USB keys.

We have evaluated the resultant systems with regards to their performance, and their integration into the workflow at our partner institution. The following sections discuss the validation and functional characteristics of INVOLVE2.

### Performance

#### Simulation environment

**Figure 5:**
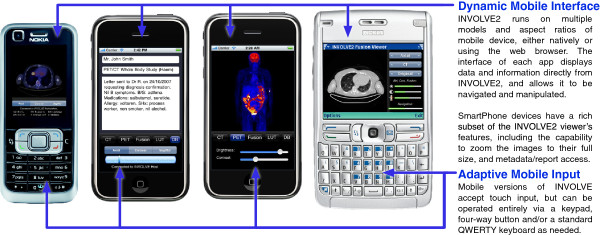
**The user interface of our mobile INVOLVE viewer, shown running on a variety of Smartphone devices.**

The INVOLVE2 suite of systems was designed to operate on average consumer devices, so as to allow its use in hospitals without dedicated infrastructure support, and enable outpatients to make use of the software directly using their home machines or available devices. In order to demonstrate the range of results for our systems’ performance in a variety of contexts, a set of Viewer, Mobile and Web performance^b^ trials were each run using two appropriate consumer-level computing devices from the upper and lower parts of the spectrum. The first of these tested the INVOLVE viewer, run on a standard desktop PC. The second tested the Mobile version of the INVOLVE viewer. Finally, the last tested the browser-based version shown on the Nokia devices in Figure 5. The results presented for each of our INVOLVE2 subsystems specify ”high-end” and ”low-end” machines as their upper and lower bounds on performance. These indications refer to computing devices with the specifications listed in Table 1.

**Table 1:** **Specifications of machines used in trial**

Trial	Low-end	High-end
Viewer performance	1.67 GHz PowerPC 7447a, 2 GB RAM, Mac OS X 10.5.8 (9L30)	2.3 GHz Intel Core i7, 8 GB RAM, Mac OS X Lion 10.7.3 (11D50b)
Mobile performance	412 MHz iPhone 3G, 128 MB RAM, iOS 4.2 (8C148)	1 GHz iPhone 4, 512 MB RAM, iOS 5.1 (9B179)
Web performance	2.16 GHz Intel Pentium T3400, 2 GB RAM, Windows Vista (32-bit)	2.2 GHz Intel Core i7, 8 GB RAM, Mac OS X Lion 10.7.3

The system used in our case study (below) remained constant throughout, namely a Mac Mini device with the following specifications: 2.66 GHz Core 2 Duo, 2 GB RAM, Mac OS 10.6.8 (10K540). No low- and high-end alternatives were necessary, as the burner represents an integrated, standalone system without the need for utilization by the end-user. The Mac Mini serves as an independent, one-piece burner system capable of handling the entire burn process.

#### INVOLVE viewer performance

**Figure 6:**
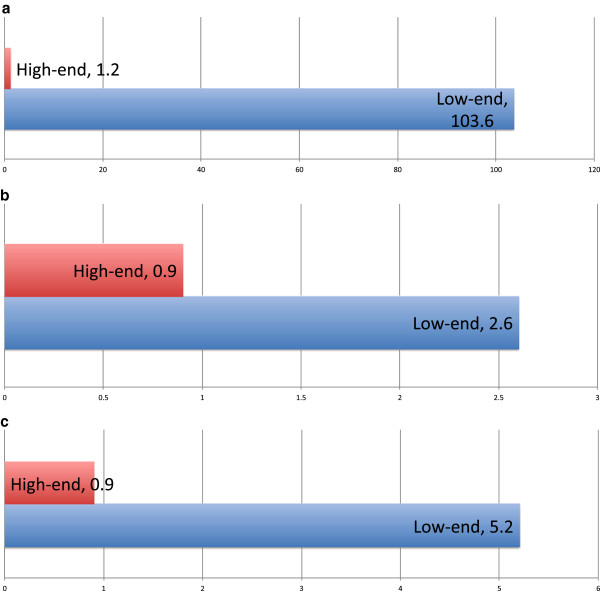
**A set of graphs showing INVOLVE2 Viewer performance when run on low- and high-end consumer-level computing devices.**

Figure 6 shows the relative consistency and rapidity of load-times for the viewer, alongside the time taken to switch one’s view to different axes (that is, view the dataset along a differing axis, which necessitates a reconfiguration of the loaded image content). As shown in the graph, load times are expressed in seconds rather than minutes, and the ability of INVOLVE2 to quickly load studies is more a function of storage medium access times than application performance.

Longer access times may be observed only where the user’s system is under significant load, or very low on memory, but even in this instance the INVOLVE2 viewer loads with sufficient rapidity to qualify as responsive under the circumstances. The highest observed load time remains under 2 minutes, which remains faster than many commercial solutions evince even under ideal conditions. In every instance - even in the case of heavy system load - manipulation, colorization and navigation of loaded data has been observed to be instantaneous.

#### Mobile INVOLVE viewer performance

**Figure 7:**
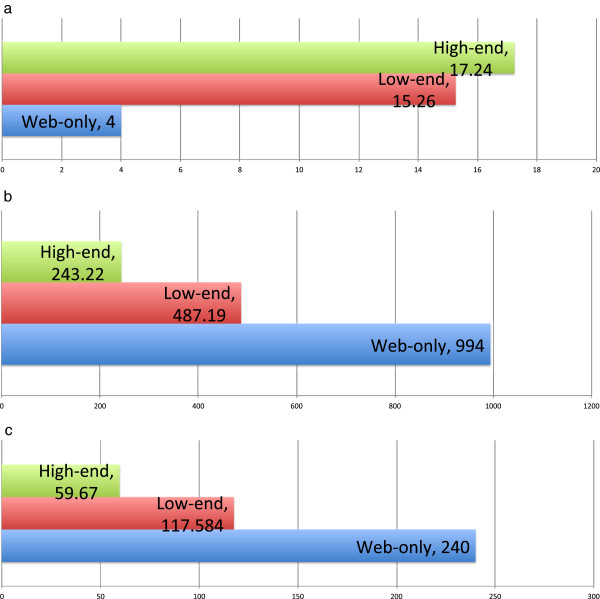
**A set of graphs showing INVOLVE2 Mobile Viewer performance when run natively on low- and high-end consumer-level computing devices, or in a mobile browser (web-only).**

As illustrated in Figure 5, the mobile INVOLVE application is capable of displaying the data and metadata stored and navigated by INVOLVE on a variety of mobile and web platforms. Our results as given in Figure 7 indicate comparable performance on both low- and high-end Smartphones, which are limited largely by bandwidth rather than processing capability, and achieve excellent frame-rates when connected to an INVOLVE viewer. A third column was added to these results indicating the performance of web-only devices - namely phones and other mobile devices which do not support apps or are not natively compatible with INVOLVE. Our test device for web-only was a Nokia E61i with a 220 MHz Dual ARM 9 processor, 64 MB RAM, and Symbian OS 9.1 (Series 60 v3.0). Web-only results indicate that browser-based use of INVOLVE software is functional but severely lacking in performance, and is unsuitable for primary use - though it may be sufficient for certain basic use-cases such as providing a condition overview to family members.

#### TAGIGEN performance

**Figure 8:**
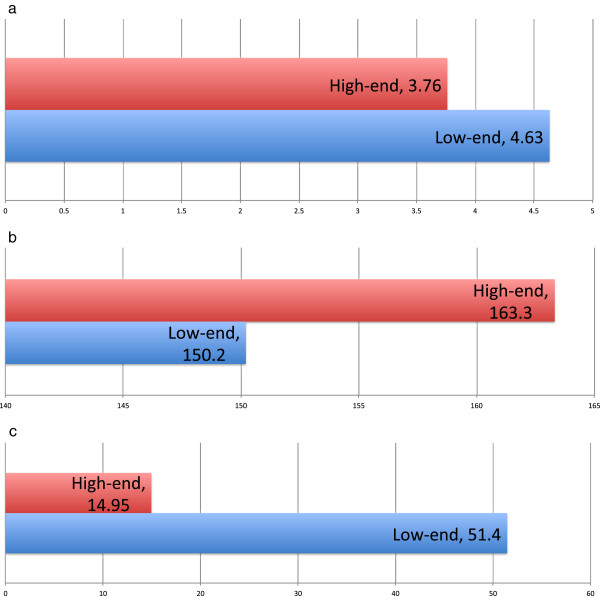
**Graphs of TAGIGEN performance when run within the Google Chrome browser on low- and high-end consumer-level computing devices.**

The use of TAGIGEN with preprocessed INVOLVE datasets exhibits significant functionality and a very low time-to-load, on both high- and low-end consumer PCs. This data is shown in Figure 8. The results of our TAGIGEN study [27] have shown that the time taken for users to navigate studies and identify desired image features using TAGIGEN is significantly reduced in comparison to conventional image browsing concepts.

### Case study: workflow implementation

We implemented INVOLVE2 in the Department of Nuclear Medicine at the Royal Prince Alfred Hospital, Sydney. As a test platform, our system was integrated into the existing hospital network infrastructure. Typically, a PET-CT study will be initiated by the patient’s primary caregiver (i.e. the referring physician), who will then be issued a report at the conclusion of the study containing the radiologist’s observations. Our system was situated such that at this final stage, an INVOLVE2 CD or USB key could be generated for potential delivery to the patient.

**Figure 9:**
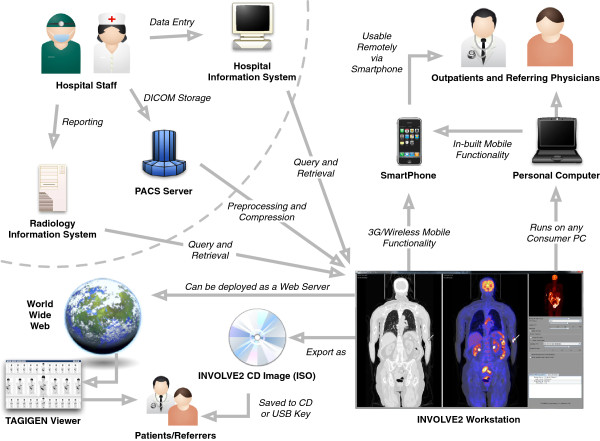
**An overview of the INVOLVE2 Workstation system’s capabilities, and stakeholders in- and out-side of the hospital who may interact with the system.**

Figure 9 shows a functional overview and exploration of the use cases of the INVOLVE2 system. Hospital staff do their job as usual, entering data into the appropriate hospital-based systems. INVOLVE2 can receive studies directly from the hospital systems and is able to combine data from these sources using its preprocessor. The INVOLVE2 viewer software can be deployed, with full functionality and imaging data, outside the hospital either by having it generate a CD or USB key containing the whole software suite and selected patient data, or over a network for mobile/Internet sharing using the in-built networking protocols.

The INVOLVE2 suite of software integrates directly into the workflow of PET-CT image acquisition and delivery. Despite the significant functionality of INVOLVE2, it introduces few steps into the actual acquisition procedure. This procedure is outlined below, highlighting which steps were introduced by the INVOLVE2 software. The average time taken for each of these steps to be completed is given in Table 2. The table also lists the typical file size of INVOLVE2 datasets and their associated TAGIGEN views, showing that the optimized formats used by INVOLVE2 are suitably sized for easy export via inexpensive CD-ROM media. Note that the times listed refer to automatic processes, and represent the average wait time until a disc can be made available, not additional time that the technician must spend per patient. How these wait-times integrate into the workflow of clinical staff is discussed below.

1. Hospital technicians and nurses put the patient in place, and begin the acquisition process. The PET-CT scanner acquires raw image data in DICOM format, which is transferred to a local PACS.

**Table 2:** **Performance and storage measurements for INVOLVE2 CD Burner workflow**

INVOLVE2 CD Burner	Average	Standard
		deviation
Size of Study	90.42 MB	3.73
Size of TAGIGEN Views	22.61 MB	2.74
Time to Send Study	38.1 seconds	9.19
Time to Preprocess	67.45 seconds	0.21
Time to Burn	133.54 seconds	11.63
Time to Generate TAGIGEN Views	7.42 seconds	1.24
Total Workflow Waiting Period	246.51 seconds	n/a
2. Minor processing is performed at the local PACS so as to cross-reference the newly acquired scan the Radiology Information System, and save thumbnails of the scan results.
3. The images are transferred via DICOM protocols to another PACS repository for current studies. This repository, in turn, forwards the images to the INVOLVE2 Preprocessor. On average, this transfer takes 38.1 seconds (see Table 2).
4. The INVOLVE2 Preprocessor performs its preprocessing tasks and begins an automated burn process automatically, providing notifications regarding its status over the hospital network where necessary. These tasks together, excepting the physical disc-burn operation, total an average waiting time of 67.45 seconds (see Table 2).
  - Once the study has been received, the INVOLVE2 Preprocessor initiates conversion of the raw DICOM files to losslessly compressed TIFF image data and a rotational MIP.
  - The preprocessor creates a file structure suitable for use by the INVOLVE Viewer, but also TAGIGEN: the same data structure used by INVOLVE2 is also organized correctly for TAGIGEN.
  - The preprocessed MIP information is encoded into a streaming video format, to allow it to be transmitted over the network to patients using either a mobile device or web browser.
  - An ISO (virtual CD image) file is created, containing the preprocessed TIFF and MIP data, the INVOLVE2 executable package and the Java Runtime Environment, which may be required on some systems to run INVOLVE.
  - The ISO is either saved to a USB key or burned to disk using the attached CD-burner hardware. If burning a CD, notifications inform radiology staff when it is necessary to manually insert/remove writable CDs, or turn them over for labelling. Presently, labelling is done via Lightscribe technology, by using the burning laser to mark the disc’s surface with identifying information. In our trials, the burn process took 133.54 seconds on average, though it will vary depending on the media and hardware used (see Table 2).
5. Once a CD or USB key of the dataset is produced, it is given to the outpatient for personal review and delivery to the referring physician. CDs run on any consumer PC, and allow the patient to:
  - View, Navigate and Manipulate their personal scan data. The viewer automatically handles all necessary load operations, requiring the user only to start an executable on the CD.
  - Share, Host and Forward their data over their home network. The SparkMed component runs as a web-host within the INVOLVE2 software, allowing remote access via mobile and web-based applications.
  - Compare, Understand and Collaborate by exploring the data alone, with specialists over the Internet, in person alongside medical staff, or in the context of their previous scans using TAGIGEN.
6. All controls should be labelled using simple English (not medical jargon) that is understandable by a layperson.
7. At the end of the day, current studies are archived by transferring from their current PACS repository to a dedicated archival PACS. This system’s contents are backed up on tape and CD automatically.

### Discussion

The above workflow bears a strong relation to similar workflows outside of our specific context, and given the increasing role of radiology in numerous clinical disciplines, it would be reasonable to consider the design of INVOLVE2 for use with other types of medical imaging. Some examples include X-ray radiographs, ultrasound, dermatological images and mammography. The majority of the INVOLVE process is agnostic to imaging content. Thus, as long as the source images are in a suitably annotated DICOM format and a simple script can be produced to inform the INVOLVE2 Preprocessor of which images are to be compressed to TIFF, and which are to be pre-rendered as streamable video, the INVOLVE process could be directly applied to any sufficiently-similar image modality. The modular structure of the INVOLVE2 viewer’s user interface allows for specialised viewing components to be added if necessary.

Any workflow built upon this process would receive the benefits of our automated CD-R burn process, staging via TAGIGEN, as well as cloud-based data transfer and display via the web or mobile devices using our SparkMed framework (the details of which are presented in [[cite]]). Given the proliferation of web-capable handheld devices such as iOS and Android smartphones and tablets in the medical enterprise [40], and the near-clinical level of clarity offered by new high-resolution mobile displays [41], this functionality alone may be sufficient reason to incorporate INVOLVE2 into these workflows. Adapting the INVOLVE2 Preprocessor to perform useful knowledge-based preprocessing, such as annotation, 3D reconstruction or window/level transforms, on a wider range of image types, could allow INVOLVE2 to institute significant improvements for imaging workflows in these related fields.

A careful balance must be struck, however, between the ease and convenience introduced by INVOLVE2’s data sharing features, and the preservation of the patient’s privacy and the integrity of their medical record. Whereas printed slides and data discs intended for use with proprietary systems can be compromised only with physical access or the system in question, INVOLVE2 datasets are self-contained and widely compatible, which warrants that greater vigilance be exercised in their distribution, as the consequences of disclosure can be far greater.

Given the always-online state of many mobile devices, and the data portability and networking features of INVOLVE2, improperly secured records could easily be posted on multimedia or social networking websites (such as YouTube, Facebook, etc.), whether deliberately, accidentally or by a malicious third party. This raises issues of privacy, security, data ownership and liability [42] which require that the risks of such disclosure be carefully weighed against the benefits, and appropriate policies, countermeasures and security software be put in place before the INVOLVE2 software is made available to the general patient population.

## Conclusions

We have described INVOLVE2, a distribution system for medical images which is effective for use both in- and out-side of the hospital, and evaluated its fitness-for-purpose in terms of its operational requirements, workflow integration, and performance on consumer hardware. INVOLVE2 consists of a cross-platform medical image display system and a suite of pre-processing components that ensure it runs quickly and effectively on a wide range of devices and across the Internet. INVOLVE2 datasets can be quickly loaded, navigated, streamed, distributed and compared, either via a standalone CD or USB key, over a network, or across the Internet. Our TAGIGEN subsystem offers a promising interface for the comparison and staging of medical scans. This interface may prove more intuitive than, and certainly makes a good adjunct to, the image-comparison solutions offered by commercial vendors. Further, most of the INVOLVE2 preprocessing workflow is automated or flows naturally into existing hospital processes, whereas our viewer’s interface remains simple for completely non-technical users to operate.

The results demonstrate that INVOLVE2 meets performance targets, supports a wide variety of consumer devices, and runs effectively across the network or from a CD or USB key. We have developed our system to a high standard using powerful, non-proprietary technologies, integrated it successfully with the network of PACS at the Royal Prince Alfred Hospital, and demonstrated its fitness-for-purpose. INVOLVE2 enables patient participation by granting easy access to complex data, and its unique distribution workflow enables fast, effective sharing.

Future work on this project will focus on expanding the annotation and reporting capabilities of INVOLVE by allowing practitioners to highlight regions of interest, and transcribe reports by voice. Further, we plan to improve the efficiency of our MIP preprocessor, and implement interface-recording and audio transmission on devices such as Smartphones so as to support the playback of doctors’ entire reporting process. We believe that this will allow us to close the gap between imaging and the communication of results even further, by allowing the radiologist who performs the initial diagnosis to directly communicate findings to the patient with minimal disruption to his or her workflow. Finally, the current implementation of INVOLVE2 is due to undergo clinical trials, whereby automatically generated INVOLVE2 CDs will be distributed to outpatients and referrers for their clinical feedback.

## Consent

All patient data used in this study was obtained with the full consent and understanding of the patients involved, and anonymised for security.

## Endnotes

^a^The burner does, however, support an optional “Growl” notification system which only runs on Mac OS X.^b^Web performance was measured using the latest version (v18.0.x.x) of the Google Chrome browser for each respective platform.
